# Religion and spiritual well-being: a qualitative exploration of perspectives of higher education faculty in Qatar and its challenge to western well-being paradigms

**DOI:** 10.3389/fpsyg.2025.1549863

**Published:** 2025-04-28

**Authors:** Hessa Al-Thani

**Affiliations:** College of Education, Qatar University, Doha, Qatar

**Keywords:** spiritual well-being, religion, education, faculty members, Qatar

## Abstract

**Introduction:**

Spiritual well-being (SWB) is a multidimensional and multifaceted concept that encompasses various spheres of human experience, including a person’s spirituality, faith, identity, values, and sense of purpose in life. This study aims to gain a contextualized understanding of how participants affiliated with different higher education institutions in Qatar perceive and experience SWB. It addresses a gap in the literature regarding the influence of cultural and religious factors on the concept of SWB in higher education, particularly in the Qatari context, where cultural factors, especially Islamic values, shape faculty well-being.

**Methods:**

An interpretive qualitative research approach was utilized, focusing on round table (focus group) discussion data obtained from a diverse group of college and university faculty members in Qatar. A purposive sampling technique was employed to recruit participants representing different institutional affiliations. Thematic analysis was used to identify recurring themes and patterns that emerged from the focus groups, viewed through the lens of socio-cultural theory.

**Results:**

Findings reveal that SWB is a very personal and subjective experience, largely influenced by religious beliefs, values, and personal life experiences. Key themes extracted from the data include the intimate relationship between religion (Islam), spirituality, and SWB; the interconnectedness between SWB and adherence to Islamic values and ethics; and the role of SWB in defining identity and in finding meaning and purpose in life.

**Discussion:**

This study contributes to socio-cultural theories of well-being by highlighting how SWB in Qatar challenges and expands the existing Western paradigms of well-being. The practical implications suggest that higher education institutions in Qatar should consider integrating the concept of SWB into faculty support practices, fostering environments that acknowledge and support the role of spirituality in well-being.

## 1 Introduction

Over the past few decades, the concept of well-being has garnered renewed and intensified attention among scientists, researchers, educators, businesses, and policymakers alike. More recently, the outbreak of the COVID-19 pandemic heightened attention to and interest in people’s health and well-being across the globe (Yaaqeib et al., 2022). Indeed, the importance of well-being to individuals, the community, and society as a whole is increasingly acknowledged, and governmental and non-governmental organizations strive to prompt employees’ well-being and provide interventions that cater to their physical, emotional, social, spiritual, and environmental well-being (Althammer et al., 2021; Gritzka et al., 2020; Pieper et al., 2019). At the local, national and regional levels, people’s well-being is gaining growing prominence as a priority in the policy agendas of many countries worldwide.

The extant literature points to myriad definitions of the construct of “well-being” and hence lack of consensus on the meaning of the term (Pollard and Lee, 2003); for, available research reveals that the boundaries between well-being, quality of life and wellness remain largely blurred (Wijngaards et al., 2021). Overall, existing scholarship dealing with well-being may be criticized as being reductionist and too focused on Western definitions and conceptualizations and, therefore, does not provide a culturally sensitive and inclusive view. Indeed, most of the work done on well-being has been conducted in the global North countries that fit the WEIRD profile, i.e., Western, Educated, Industrialized, Rich, and Democratic nations (Abma et al., 2019; Hendriks et al., 2019). By contrast, an evident dearth of useful theories about well-being, specifically SWB, in countries of the global South.

Looking at well-being research carried out in Arab-Muslim societies, it appears that this area is still largely understudied (Musa, 2015), thus resulting in an insufficient understanding of the topic in a non-Western, Arab-Muslim context. When investigating well-being in Islam, it is crucial to be mindful of context-specific limitations that should be taken into account. Herein lies and hence the importance of adopting an inclusive approach that recognizes the diversity of Muslim societies and the various demographic, economic, social, cultural, and political differences that exist within and between them. Equally important is the need to be cautioned against subjectivity, cultural bias and reliance on Western-centric evaluation measures, which may not properly capture the customs, traditions, and values espoused in Islam.

In Qatar, as an Arab-Muslim country, significant emphasis is placed on spirituality as an essential component of individual and societal well-being (Abu-El-Noor and Abu-El-Noor, 2021). Deeply rooted in Islamic values, well-being in Qatar is closely linked with religiosity and community values (Al Shamari and O’Hara, 2024; Abdel-Khalek, 2014b). Islam shapes various aspects of daily life, influencing personal resilience, social interactions, and overall life satisfaction (Ghuloum et al., 2024). The Qatari government and local institutions actively promote well-being through policies and initiatives that integrate spiritual, social, and psychological support. Programs that emphasize the role of faith, values of truth, honesty, justice, and moral rectitude fostering social cohesion, and enhancing mental and SWB have gained increasing attention in recent years in Qatar (Al-Wattary et al., 2025; Al-Wattary, 2022). Given this strong cultural and religious framework, exploring SWB in Qatar provides a valuable perspective that challenges dominant Western conceptualizations and contributes to a more understanding of the construct.

Our study employs socio-cultural theory to explore SWB among faculty members in Qatar, acknowledging its emphasis on social and cultural factors. However, we recognize the value of alternative frameworks such as Islamic epistemology, phenomenology, and postcolonial psychology. Islamic epistemology offers a unique perspective on spiritual growth and intuition, while phenomenology delves into subjective experiences. Postcolonial psychology critiques Western-centric models, emphasizing cultural diversity. Despite these alternatives, socio-cultural theory corresponds best to our focus on social context. An Islamic perspective enriches these models by integrating spiritual and moral growth, aligning with Islamic thinkers like Al-Ghazali, who emphasized soul purification as a path to well-being. By incorporating this perspective, our study provides a more holistic understanding of SWB, challenging dominant Western paradigms and contributing to a culturally nuanced view of well-being in Arab-Muslim contexts.

Findings derived from the current research should provide data-driven evidence that will guide the design of relevant educational programs, inform the development of social supports, and advance the promotion of social and cultural cohesion and understandings between and within groups and communities in Qatar and beyond. Ultimately, the conclusions drawn from this study will shed light on the need for promoting programs and activities that serve the emotional, intellectual, physical, social, and spiritual needs of individuals in order to empower their inner abilities to face life challenges. It is hoped that this will also contribute to creating leveraging opportunities to educate people’s Heads, Hearts and Hands as a way of enabling them to take pride in their beliefs, customs, values, traditions, and their belonging.

This paper is organized as follows. The next section contextualizes the current study’s research problem providing relevant background information. It offers a review of the available literature that has addressed the concept of SWB from Western as well as Islamic perspectives. This is followed by a detailed description of the methodology used in this study, including the research design, participants, data collection and the mode of data analysis adopted. In the section that follows, the study’s results are presented, focusing specifically on how social and cultural influences shape the concept of SWB. Finally, the paper concludes with a discussion of the findings and provides recommendations for future research.

## 2 Review of literature

The existing literature shows that “well-being” is a largely contested term (Cooke et al., 2016) and a concept that is large in scope, encompassing various aspects of a person’s experience of health, happiness, and sense of safety, as well as a feeling of belonging, achievement, and success. Demonstrably, there is no consensus on a singular definition of well-being (Kowalski and Loretto, 2017; Nelson et al., 2015), which is generally understood as referring to an individual’s personal satisfaction and experience of happiness, including mental and emotional health. Thus, myriad definitions have been proposed in past and recent scholarship, viewing the concept of well-being through demographic, disciplinary, social and cultural lenses (Camfield and Skevington, 2008; Hanlon and Carlisle, 2008).

Building on previous conceptualizations of well-being, Bartels et al. (2019), Mans et al. (2021), and Psaila (2019) define well-being as a multi-dimensional and multi-faceted construct incorporating mental, physical, psychological, social and cultural dimensions (Psaila, 2019). In this study, well-being is conceptualized as a multi-dimensional concept, encompassing emotional, intellectual, physical, social, and spiritual dimensions (Schulte et al., 2015; Trudel-Fitzgerald et al., 2019). For the purpose of the present study, we focus SWB, which comprises personal and subjective domains, and what lies beyond that which is material or physical; this involves the inner self, beliefs, and values as well as what is religious or non-religious.

According to the World Health Organization (2004), well-being is a state in which “the individual realizes his or her own abilities, can cope with the normal stresses of life, can work productively and fruitfully, and is able to make a contribution to his or her community” (World Health Organization, 2004, p. 10). Generally, strong associations have been found between well-being and a host of positive outcomes for individuals and society. There is ample evidence to suggest solid links between higher levels of well-being and successful achievement in different spheres of life (Martela and Sheldon, 2019; Pleeging et al., 2021). For example, research has shown positive relations between well-being and health (Abma et al., 2019; Otto et al., 2021; Ross et al., 2020); community and social support (Atkinson et al., 2020; Taylor, 2020; Macdonald et al., 2018); workplace environment (Althammer et al., 2021; Gritzka et al., 2020; Pieper et al., 2019); and life satisfaction (Hendriks et al., 2019; Kim et al., 2021; Emerson et al., 2021).

Considerable international research has explored diverse dimensions of well-being, particularly those that concentrate on mental, psychological, and physical health. At the same time, interest in religiosity and SWB is growing in importance (Abdel-Khalek, 2019). More specifically, the last few decades have witnessed a surge in research on the spiritual dimension of well-being, calling for the use of a multi-dimensional and multi-disciplinary approach when studying well-being (Psaila, 2019). As is pointed out by Swarbrick (2006), well-being is “holistic and multi-dimensional, and includes physical, psychological, intellectual, social, environmental, and spiritual dimensions” (p. 311).

Underpinning research on well-being are two theoretical frameworks: subjective and objective well-being (Ross et al., 2020). The subjective approach emphasizes personal experiences and individual fulfillment, including eudaemonic well-being (e.g., finding meaning in life and experiencing personal growth) and hedonic well-being (e.g., feeling happy and satisfied with one’s life) (Ross et al., 2020). In contrast, the objective approach views well-being in terms of quality of life indicators, such as material resources (e.g., income, food, and housing) and social attributes (e.g., education, health, political voice, social networks, and connections) (Ross et al., 2020). While the subjective and objective frameworks provide a broad understanding of well-being, a more focused exploration of psychological well-being reveals its complex and multi-dimensional nature.

Psychological well-being, a crucial component of overall well-being, has been widely explored through various theoretical perspectives. Ryff (1989) conceptualized psychological well-being as comprising six dimensions, i.e., autonomy, environmental mastery, personal growth, positive relations with others, purpose in life, and self-acceptance. Diener (1984) and Lyubomirsky (2001) further emphasized the role of positive emotions and life satisfaction in shaping psychological well-being, highlighting the association between cognitive evaluations and emotional experiences. These perspectives underline the importance of both personal and social factors in enhancing well-being, reinforcing the need for a holistic approach to understanding its determinants.

Building on these perspectives, this study adopts a multi-dimensional view of well-being, recognizing both its subjective (personal fulfillment, life satisfaction) and objective (quality of life, social support) components. The particular focus is on SWB that aligns closely with eudaimonic well-being, as it emphasizes meaning, values, and inner self-awareness rather than mere pleasure or external success (i.e., goal achievement or life satisfaction). By integrating these conceptualizations, the study operationalizes SWB through emotional, intellectual, physical, social, and spiritual dimensions, reflecting its multifarious nature.

### 2.1 SWB

Prior research reveals the existence of positive relationships between well-being, on the one hand, and spirituality (Heintzman, 2020; Villani et al., 2019), religiosity (Ardelt and Ferrari, 2019; Gross-Manos and Massarwi, 2022) and SWB (Bożek et al., 2020; Johnson and Carter, 2020). There is ample evidence to suggest a connection between different facets of spirituality and one’s overall life contentment. For example, life satisfaction appears to be associated with spiritual beliefs (Sargent, 2015), religious practice or personal spirituality (Sharajabad et al., 2017), and spiritual (Alorani and Alradaydeh, 2018).

Whereas evidence of research involving associations between religious beliefs and practices and SWB is copious, the bulk of these studies is based in Western countries, pointing to a paucity of literature that addresses SWB in Arab-Islamic environments. For the purpose of this study, the focus will specifically be on SWB. The concept of SWB is multi-dimensional and hard to define, mainly due to the relationship between spirituality and religion (Psaila, 2019). Over a century ago, Durant Drake (1916, p. 244) explained the meaning of “spirituality” and its relation to “religion” as follows:

The disposition of the heart and will, through which a man comes to care for the highest things and to live in gentleness and inward calm above the surface aspects and accidents of life, we call, in its inner nature, spirituality; when it is embodied in outward forms and institutions and spread among whole communities, we call it a religion.

Islam considers well-being (SWB) as a holistic concept encompassing spiritual, moral, physical, emotional, and social dimensions, guided by the Qur’an and Sunnah (Bensaid, 2018). It emphasizes the interconnectedness of mind, body, and environment, viewing SWB as essential for a fulfilling life in this world and the hereafter (Abdul-Rahman, 2017; Psaila, 2019). Spirituality in Islam stems from faith, good deeds, knowledge, and righteousness, contributing to a happy life (Amiruddin et al., 2021). The Qur’an states that those who do good deeds and believe will be rewarded with a good life (Surat Al-Nahl: 97) (Abdel Haleem, 2004). Islamic spirituality involves a relationship with Allah that influences self-worth, meaning, and connection with others (Ghobary Bonab et al., 2013).

Ibn Hazm (994–1064) emphasized devotion, faith, and moral integrity as central to spiritual fulfillment, highlighting piety (Taqwa) and mindfulness of Allah as fundamental to SWB (Abdul-Rahman, 2017). He believed seeking meaning helps one overcome existential crises. In the same vein, Al-Ghazali (1058–1111) advocated for purifying the soul by eliminating vices like envy and cultivating virtues like humility. Ibn Al-Qayyim Al-Jawziyyah (1292–1350) stressed developing a deep love for Allah combined with devotion and obedience to achieve closeness to the Divine and SWB. He also emphasized detachment from worldly desires.

Spirituality in Islam can be understood through the three “Hs”: Head, Heart, and Hand, which represent the cognitive, affective, and behavioral dimensions of a Muslim’s spiritual journey. The Head symbolizes the pursuit of knowledge and wisdom, encouraging Muslims to seek understanding of Islamic teachings, the Qur’an, and the life of the Prophet Muhammad (PBUH). The Heart reflects devotion and reverence for Allah, emphasizing sincerity in worship, love, and gratitude toward God. The Hand represents the behavioral aspect, where faith is translated into righteous actions. This includes performing daily prayers, charity, and living in accordance with Islamic principles in everyday life. Together, these three elements guide a Muslim in cultivating a balanced spiritual life, where knowledge, devotion, and action work in harmony to strengthen one’s relationship with Allah and lead to a life of moral integrity and spiritual fulfillment.

Research suggests that both personal and professional factors significantly impact faculty members’ mental health and job satisfaction, highlighting the critical role of policies in alleviating stressors related to work-life balance (Ceri and Cicek, 2021). When comparing Qatar’s approach to faculty well-being with that of other Islamic countries, it is essential to explore the legal and policy frameworks supporting these initiatives. In alignment with Qatar National Vision 2030, which emphasizes the development of a sustainable and thriving society, mental health has been recognized as a crucial component of overall well-being. The Ministry of Public Health in Qatar underscores the importance of mental health alongside physical health, highlighting its impact on how individuals feel, think, and interact with others (National Mental Health Program, 2018). According to the Ministry, good mental health is essential for coping with life’s stresses, maintaining positive relationships, and contributing effectively to society. It encompasses positive self-esteem, emotional regulation, and productivity.

To support mental well-being, Qatar encourages its citizens and residents to adopt healthy habits, such as eating balanced diets, staying physically active, nurturing social connections, obtaining adequate sleep, and engaging in relaxation techniques. The country’s legal framework, including the Mental Health Law 16 of 2016, further supports these efforts by providing structured guidelines for mental health care, ensuring that faculty and all citizens have access to essential support services (Weill Cornell Medicine-Qatar, 2021). This focus on mental health is in line with the broader goals of Qatar National Vision 2030, which aims to promote the well-being of individuals while fostering a productive and resilient workforce to drive the country’s long-term development. Some comparisons with other countries’ faculty wellbeing programs are as follows.

Saudi Arabia’s Vision 2030 educational reforms prioritize faculty well-being through different support systems, with institutions offering wellness initiatives, including sports facilities and psychological support, in line with the national goal of advancing public health (Asfahani, 2024). The Vision 2030 initiative aims to transform the kingdom’s education system by integrating well-being into the broader national health and social development goals. By promoting environments that support physical and mental health, public and private institutions are better able to create productive, resilient, and motivated individuals, which ultimately enhances the quality of life. For example, Saudi Arabian universities, including Prince Sultan University, are establishing wellness programs that offer a range of services from sports and fitness to mental health counseling, ensuring that faculty are supported both professionally and personally (Al-Ghalib and Salim, 2018).

To support mental well-being, Turkey encourages its citizens and residents to adopt healthy habits, such as maintaining balanced diets, staying physically active, nurturing social connections, obtaining sufficient sleep, and engaging in relaxation practices. The country’s legal framework, including the Mental Health Law No. 2827 of 2004, further supports these efforts by establishing structured guidelines for mental health care, ensuring that individuals across the nation have access to essential services and support (Turkish Ministry of Health, 2021). This emphasis on mental health aligns with the broader goals of Turkey’s 2023 Vision, which seeks to improve the overall well-being of its population while fostering a productive and resilient workforce for the country’s continued growth and development.

In Malaysia, well-being programs are embedded within national policies designed to enhance resilience and job satisfaction. Citizens and residents are encouraged to adopt healthy habits, such as maintaining a balanced diet, engaging diet, engaging in regular physical activity, fostering social connections, ensuring adequate sleep, and practicing relaxation techniques. The country’s legal structure, including the Mental Health Act 2001, provides guidelines for mental health care, ensuring that all individuals have access to essential support services. Additionally, Malaysia’s policies on mental health, as outlined in the National Mental Health Policy 2013, focus on promoting mental well-being, preventing mental health disorders, and improving mental health services for all populations (Ministry of Health Malaysia, 2013). This emphasis on mental health aligns with the broader objectives of Malaysia’s Vision 2020 and the Shared Prosperity Vision 2030, which aim to build a harmonious, healthy, and productive society while ensuring the nation’s long-term development.

### 2.2 Measurement of well-being

A wide range of conceptualizations of well-being and the proliferation of approaches to this concept has led to confusion regarding how to properly define and measure this complex construct. Similar to well-being, measures of SWB comprise objective measures, which mostly refer to the standard of living, and subjective measures, which capture psychological, social, and spiritual aspects and are based on cognitive and affective judgments individuals make about their lives (Schulte et al., 2015). The measurement of SWB is a complex task and, as such, presents many challenges. These challenges reveal the need for a more comprehensive approach that takes into account the different dimensions of SWB and the complex social and cultural contexts in which it occurs. Major challenges to the measurement of SWB include:

1. Subjectivity: wellbeing is subjective, and individuals may have different perceptions of what it means to them. There is no universal or agreed-upon definition of wellbeing.
2. Multidimensionality: well-being is a multidimensional concept that covers various aspects of life (physical health, emotions, social relationships, material resources, etc.). Measuring all these dimensions comprehensively is a challenge, as they are interrelated and can influence each other.
3. Lack of consensus: there is no universal consensus on what constitutes well-being: different societies, cultures and communities hold different views and have different values regarding what constitutes a good life.
4. Response biases: individuals may not always give accurate or truthful responses when asked about their well-being. They may feel pressure to describe themselves in a positive manner, or they may not want to talk about their feelings and experiences or they may not be fully aware of these feelings and experiences.

### 2.3 Approaches to measuring wellbeing

Two general approaches to measuring wellbeing: objectives and subjective assessments. The first, objective (quantifiable) assessments of well-being, draws on measurable and observable factors (e.g., education level, employment status, income, physical health, life expectancy). Objective measures can provide important insights into the factors that contribute to well-being. The second, subjective assessments of well-being, refers to individuals’ feelings, perceptions and evaluations of their own well-being, i.e., the assessment of an individual’s well-being as perceived and reported by the individual themselves. Subjective assessments are largely personal and can vary widely from one individual to another. They are often based on self-reported measures (e.g., happiness, life satisfaction, overall quality of life) and are influenced by a wide range of factors (cultural background, individual values, personal experiences).

## 3 Problem statement

While spirituality and spiritual care in the Arab world have received considerable attention (Abu-El-Noor and Abu-El-Noor, 2021), there is a notable gap in the literature exploring the concept of SWB, particularly from the perspective of educators in Qatar. Although some studies have examined the relationship between religiosity and health in Qatar (Ouanes et al., 2021; Al-Mansouri, 2023; Al Shamari and O’Hara, 2024), the studies by Abdel-Khalek (2014a) highlighted strong associations between subjective well-being, health, and religiosity. Despite the significance of SWB, a few studies in Qatar have focused on incorporating spiritual values into educational programs to enhance spiritual well-being (Al-Wattery, 2022).

Thus, this study aims to explore the perspectives and common understandings of the concept of SWB among local community members representing economic, political, social, cultural, and religious groups in Qatar. The purpose is to gain an informed understanding of SWB and develop an indigenous and contextualized Arab-Islamic perspective of this concept. The main goals of the study were to provide a conceptualization of well-being informed by the local and regional context, and explore different understandings of well-being and compare these understandings to the extant literature. Participants were asked questions about their definition and conceptualization of SWB and were prompted to reflect on their career experiences and practices. The questions guiding this study, therefore, are:

1. How is the concept of SWB perceived by college/university faculty members in Qatar?
2. What are the main themes underlying the opinions, experiences, and perspectives of college/university faculty members regarding SWB?
3. What is the likely influence of faculty members’ institutional affiliation (college/university) on their perspectives regarding SWB?

## 4 Research methodology

This study uses a qualitative interpretive method to gain insight into the nature of SWB in the context of Qatar. It employs socio-cultural theory, as conceptualized by Vygotsky and Cole (1978) and draws on grounded theory (Glaser and Strauss, 1967; Strauss and Corbin, 1990). In so doing, it adopts a view of discourse as social practice (Fairclough, 2001, 2013). Using the interpretive methodology, data was collected using round-table discussions, which were organized as a series of focus groups (Kitzinger, 1995). Because the intent was to explore cross-sector perspectives and stimulate the exchange of ideas, a deliberate interactive mode of qualitative data collection was deemed most suitable for this research.

Thematic analysis, guided by the principles of Braun and Clarke (2006), served as our primary data analysis method for identifying recurring themes related to participants’ perspectives on SWB. Socio-cultural theory functioned as a lens through which we interpreted these themes, recognizing the influence of cultural context, values, and social interactions on participants’ experiences of SWB within the Qatari context.

Drawing on cultural theory, we argue that it is useful to see participants’ narratives as one aspect of the manifestation of cultural norms and values and as collective meaning-making. Demonstrably, “culture surely “specifies” the prevailing images, themes, plots, and meanings that life stories may exhibit within a particular stratum or grouping of human beings” (McAdams, 2019, p. 11). As Fraser (2004, p. 180) notes, “Whether it is in the general community, workplace or home, culture shapes how individuals envisage their world and speak about their places in it.” Because discourses are “ways of being in the world” and “forms of life” that are socially situated identities (Gee and Gee, 2007, p. 3), spoken language can be interpreted to disclose social and cultural information.

### 4.1 Participant selection: purposive sampling

Given the study’s aim to explore the perspectives of higher education faculty members in Qatar on spiritual well-being, purposive sampling was chosen to ensure the inclusion of participants with diverse backgrounds, institutional affiliations, and experiences relevant to the research topic. This approach allowed for the selection of information-rich cases that could provide a nuanced understanding of SWB in the Qatari context. The researchers facilitated 12 round-table discussions, each with a designated moderator and two note-takers who have a sound background in research. All round tables consisted of 8–12 participants, including both male and female residents of Qatar. The discussions consisted of three 30-min discussion sessions, with 15-min interval breaks following the first and second sessions. Participants in the 12 round-table discussions were members of Qatar’s local community, including policymakers, business owners, service providers, healthcare employees, educators, researchers, teachers, students, religious scholars, charity organization representatives, and other service providers. In total, 74 participants from different backgrounds took part in the round-table discussions (Table 1).

**Table 1:** Summary of round-table discussion participants.

Number of participants	74
Number of round-table discussions	12
Participants per round table	8–12
Gender composition	35 Males and 39 females
Nationality	Residents of Qatar
Discussion format	3 × 30-min sessions with 15-min breaks
Participant backgrounds	4 Policymakers
	3 Business owners
	4 Service providers
	5 Healthcare employees
	11 Educators
	18 Teachers
	11 Researchers
	8 Students
	3 Religious scholars
	3 Charity organization representatives
	4 Other service providers
Staff per round table	1 moderator, 2 note-takers

Of the 12 round-table discussions, 11 were conducted in Arabic, the native language of most of the participants. Only one round-table discussion involved speakers of English as a first language and was conducted in English. To ensure the validity of the interview questions, a pilot study was conducted in April 2023, involving 13 community members representing different sectors of society. The questions were revised, developed further, and refined based on the feedback obtained from the pilot.

Pilot interviews were instrumental in ensuring the clarity, relevance, and cultural sensitivity of the study’s instrument. Specifically, based on feedback from pilot participants, several revisions were implemented and the original phrasing of some questions was found to be ambiguous, leading us to reword them for greater clarity and to avoid possible misinterpretations. We also adjusted the order and flow of the questions based on the pilot interviews to create a more natural and engaging conversation. These revisions were crucial in strengthening the validity and reliability of our findings.

In selecting participants for this research, the technique of purposeful sampling was used (Patton, 2005) which is a useful tool that allows the choice of “information-rich cases for study in depth. Information-rich cases are those from which one can learn a great deal about issues of central importance to the purpose of the inquiry, thus the term purposeful sampling. Studying information-rich cases yields insights and in-depth understanding rather than empirical generalizations” (Patton, 2002, p. 273). According to Guetterman (2015), purposeful sampling “is not a matter of representative opinions, but a matter of information richness” and the target of qualitative researchers is to “explain, describe, and interpret” (p. 3).

In line with this study’s aims, an initial list of potential participants was prepared. The list was then populated with names of residents selected from a variety of sectors and settings. Upon identifying the names of participants, high-profile figures (government ministers and policymakers, members of the Shura Council (Qatar’s parliament), and prominent business owners) were contacted by phone, and invitations were sent via mail to the other participants. A total of 90 individuals were invited, of whom 11 did not respond to the researchers’ invitations. A further 5 agreed to participate but did not attend on the day, due to extenuating circumstances.

### 4.2 Setting and data collection

The purpose of the data collection process was to decipher the defining characteristics of SWB and participants’ experiences and interpretations of this concept “in the context of the lived world” (Palmer et al., 2010, p. 99). The round-table discussions were informal, unstructured, and open-ended. As categories began to emerge from the data, the sessions became more focused. All participants were contacted via email and phone calls to see if they would be interested in taking part in the study. Those who agreed to participate were instructed to read and digitally sign an informed consent form that specified the aim of the study and stated that participation in the study was voluntary.

The series of round-table discussions took place from May 28 to June 1, 2023, in a large spacious room set up in boardroom style at the Student Center, Qatar University. Each day was dedicated to one of five pillars as follows:

- Day 1 (May 28): SWB.
- Day 2 (May 29): emotional well-being.
- Day 3 (May 30): intellectual well-being.
- Day 4 (May 31): social well-being.
- Day 5 (June 1): physical well-being.

Given the large amount of data gathered, in this study, the researchers focused exclusively on data related to SWB obtained from 28 higher education college/university faculty members. The aim is to look into the other pillars in other research studies.

Overall, approximately 90 min of participant data were gathered. Each session began with a brief introduction of the topic followed by subsequent discussions among participants. Prior to the event, moderators attended online training workshops provided by a UK-based expert the week preceding the event. A snacks-and-beverages break was offered between the first and second sessions as well as the second and third sessions.

Hand-written notes were taken by note-takers using a template prepared prior to the event. The template contained information on the date of the round-table discussion, the number of participants (broken down by sector), the names of the moderators and note-takers, and a brief summary of participants’ backgrounds. It also included a summary of the comments on the discussions and pertinent quotes from the participants.

### 4.3 Data analysis

We employed a semi-structured focus group approach to examine faculty members’ understanding and experiences of SWB in Qatar’s higher education context. This method facilitated a structured exploration of key themes while allowing flexibility to probe emergent topics raised by participants. To guide the discussions, we developed an initial set of open-ended questions aimed at eliciting participants’ personal perspectives and experiences. These questions addressed core areas such as defining spiritual well-being, identifying associated challenges and opportunities, and understanding its implications for daily life and work. As data collection progressed, our questioning evolved in response to emerging themes. For example, participants highlighted work-life balance as a significant concern, prompting follow-up questions on managing professional demands while maintaining well-being. Additional discussions explored the role of social connections, the ways in which work contributes to a sense of purpose and meaning, and the coping mechanisms faculty employ to manage stress. By refining our approach, we captured insights that were not initially anticipated. This enhanced the depth and rigor of our methodology, ensuring a comprehensive understanding of the faculty’s experiences and perceptions of SWB.

Findings were compared and contrasted using documentation from the 28 individuals who participated in the SWB round-table discussions. To improve consistency and reliability, all notes and coded data were checked for accuracy, analysis and identification of key themes by two research professors. The data was then uploaded into the reference management and data analysis MAXQDA software for further analysis. This helped to examine the perspectives, themes, and discourse narratives pertaining to SWB.

The translation of the 11 Arabic focus group data involved the following steps. Firstly, original focus group discussions were transcribed verbatim, with each transcript given an identifier in order to ensure traceability and organization. Secondly, transcripts of the Arabic data were sent to experienced Arabic-English translators proficient in both languages to translate the transcripts from Arabic into English. Thirdly, to preserve the original meaning and ensure that the translated content reflected the participants’ responses, all translated transcripts were reviewed by the researchers and two faculty members. This helped to determine a few misinterpretations and inconsistencies in the translations.

Notes from the round-table discussions, together with the participant observation notes, were transcribed to facilitate coding and then thematically analyzed with the qualitative analysis software. The data provided a valuable opportunity to analyze multiple voices heard at one sitting. Themes were identified as they emerged from the analysis and to derive categories from the analysis in a bottom-up, inductive method (Patton, 2002), a data-driven and code-oriented approach was utilized. Thus, multiple readings of the data were carried out. First, the data was read with the aim of coding and preparing transcripts verbatim in order to capture the dialogues, expressions, and nuances of participants. The data was then read and re-read to identify meaningful themes and patterns. Quotes and examples were then integrated into various themes to support findings.

## 5 Findings

As was pointed out earlier, the present study employed a narrative mode of inquiry, which is “a phenomenological qualitative research methodology [that] examines individual human experiences—stories” (Ford, 2020, p. 235). This study’s analysis draws on the view of narratives as life stories, delving into “how people make sense of life through the act of narrating” (Rodríguez-Dorans and Jacobs, 2020, p. 612). In this sense, narratives constitute a type of “storying stories” (McCormack, 2004, p. 2019) and thus “depict social phenomena through people’s stories of everyday life experience” and serve to “to reflect, interpret, and communicate narrated experiences” (Rodríguez-Dorans and Jacobs, 2020, p. 613).

The findings of this study are presented under the overarching theme of college/university faculty’s perceptions and understandings of SWB and, in so doing, the researchers dig into the major themes that the data generated. Here, it has to be noted that a challenge that the researchers encountered in analyzing the data was whether to present and describe this data by academic discipline, theme, or a combination of both. The decision to analyze and organize the data using a combination of both approaches stems from the need to generate a holistic understanding of SWB and ascertain a comprehensive view of related overarching concepts. This combination also served to look at (a) diverse SWB perspectives and commonalities or differences across disciplines and (b) the unique and contextualized perceptions and definitions of the term within each distinct discipline and understand discipline-specific nuanced meanings.

### 5.1 How is the concept of SWB perceived by college/university faculty?

This study’s analysis revealed a range of key themes and patterns associated with SWB. Viewed from the perspective of college/university faculty, the findings indicate variations in participants’ views and perceptions of SWB. Narrative accounts by participants indicated that religion (Islam) plays a very significant role in the way SWB is perceived, understood, and experienced. Therefore, participants’ narratives and reflections should be understood as being nested within complex social and contextual relationships.

Underlying the views and perceptions of the participants are four salient overarching themes that emerged from the data; these are presented as follows below: (a) SWB, spirituality, and faith; (b) SWB, morality, and Islamic values; and (c) SWB, identity and purpose in life. Each of these themes was treated as comprising personal stories and narratives. Contemplating participants’ narrative accounts shows diverse perceptions of the definition and significance of SWB, which are rooted in contextualized backgrounds and interpreted in very subjective and personal terms. A common thread running through the descriptions provided by participants is the closely intertwined relationship between SWB and religion, spirituality, and personal growth and development.

#### 5.1.1 SWB, spirituality and faith (Islam)

Participants’ narratives generally highlighted the significant role that religion (Islam) plays in determining SWB, perhaps signifying that religious beliefs shape their SWB. For example, participant 16, a male faculty at the College of Islamic Law (Shari’ah), stated that “Religion is what influences our SWB. It even determines our well-being.” In describing SWB, participants often used subjective statements that embody the relationship between religion, attitudes toward spirituality, and their personal well-being. Participants frequently referred to their religion as “a core component of a person’s life and as an integral component of people’s overall well-being,” reflecting their relationship with their creator (Allah).

Participant 7 (female faculty, College of Education, Qatar University):

SWB reflects our relationship with our Creator and with those around us. Compared to other forms of well-being, SWB is the most important. In fact, it is at the heart of all other pillars of well-being [emotional, intellectual, social, and physical well-being].

A central element that the narratives yielded is participants’ belief in a higher power and a strong sense of faith, highlighting a strong connection with their Creator (Allah) and their religion (Islam). Contemplating the influence of religion and the Divine on her life, participant 2 (female faculty, University of Doha for Science and Technology), commented:

Our spiritual happiness and prosperity are determined by our belief that there is a power greater than all of us and that is Allah. As educators, we need help learners to understand the meaning of life and what is the value system that governs our behavior.

Another (participant 10, a male faculty at the College of Business and Economics, Qatar University) contended that:

All our experiences, practices, and perspectives must originate from our religion and from our special relationship with our Creator; they must be based on harmony with our innate nature, that is, the principle of spiritual harmony between us, the universe, and other people. We must educate our children to aim at meeting goals that are consistent with the Shari’a (Islamic law).

Narrative extracts such as these show a strong sense of faith that is deeply rooted in religion and the Divine. Participants often asserted that Islam helps them to find answers to many of “life’s questions,” as Participant 24 (a male faculty, the College of Shari’ah, Qatar University), suggested. Another participant (P 3, a male faculty at Doha University of Science and Technology) stated the importance of attachment to Allah in strengthening one’s spirituality, noting the need to consolidate one’s relationship with Him:

It’s crucial for us to know Allah very well so that our connection with Him is strong. This is essential. This is also important for the purification of the human soul as it makes people able to control themselves and their desires and material pleasures.

The narrative transcripts further emphasized consistent and frequent references to the principles of Islam as a guiding framework in people’s lives and for their personal development. The preceding analysis demonstrates that SWB refers to a sense of meaning and purpose in life, both of which are derived from spiritual beliefs and experiences. This is a point the following section focuses on.

### 5.2 What are the main themes underlying the opinions, experiences, and perspectives of college/university faculty members regarding SWB?

#### 5.2.1 SWB, morality, and Islamic values

Another theme identified in the data is the intertwined relationship between SWB and adherence to Islamic ethics and the principles of good moral standing. Arguably, religious or spiritual beliefs may be indicative of people’s moral and prosocial behavior, as participants’ narratives revealed. For instance, participant 13 (a male faculty at the Community College) remarked, “Our morals and behaviors are grounded in our faith and this shows the importance of living in accordance with our beliefs.” A common understanding that emerged from the narratives was that morality in Islam is deeply rooted in the principles and teachings of the Quran and the Hadith (Prophet Mohamed’s sayings), which provide the principles guiding righteous behavior and ethical conduct. As participant 5 (a female faculty at Doha University of Science and Technology) remarked,

Islam attaches a lot of importance to spirituality as there are many fundamentals that strengthen this, as is clearly stated in the Qur’an and our Prophet’s sayings. For example, the five pillars of Islam emphasize worship, and all aspects of faith stress the importance of ethics and morals.

In the view of another (participant 21, a male faculty, College of Arts and Sciences, Qatar University),

Islamic teachings emphasize the importance of moral values with the aim of cultivating an Islamic character and fostering a harmonious community that embraces morally upright individuals.

For Participant 16 (male faculty, College of Shari’ah, Qatar University): “Tolerance and the right understanding of religion lead to righteousness and sound morals in one’s life.” Similarly, participant 9, a College of Education male faculty at Qatar University, had this to say:

Adhering to the principles of Islam is essential because our religion guides the different aspects of our behavior. This is why we have to live in accordance with Islamic ethics and moral values. This will in turn help us gain a sense of contentment and inner peace and prevent us from indulging in desires, worldly or moral corruption.

The promotion of moral values and the standards of right and wrong within society is viewed by many respondents as integral to SWB. In the words of Participant 11 (a male faculty at the College of Business and Economics, Qatar University), “Faith provides the tools and coping strategies for handling many of our social ills and immorality.” A similar perspective was offered by Participant 2 (a female faculty at the College of Shari’ah, Qatar University), who expressed a firm belief in faith seen as a means of abandoning sins and maintaining morality, perceived as being deeply anchored in “our religion, Islam.” In the view of participant 17, a College of Engineering male faculty at Qatar University,

Islam teaches us the proper way to behave and deal with others and the need to uphold Islamic values and moral principles. This is a religious and moral duty that we Muslims should honor. We have many, many examples to emulate from the Qur’an and the sayings of Prophet Mohamed (peace be upon him).

A similar view was corroborated by participant 20, a female faculty at the College of Health Sciences, Qatar University, who indicated that “At the core of our religious beliefs are the ethical values and morals that show us how to distinguish between right and wrong in society.”

It was also obvious from participants’ narratives that SWB involves a wide spectrum of moral values, principles, and ideals within the local community. Examples that stand out include honesty and truthfulness, fairness, personal integrity, respect for others, tolerance, and understanding, etc. These values and ideals were reported to govern people’s behavior, guide their decisions, and help them to make informed choices that align with their spiritual beliefs. For instance, participant 12, a female faculty from the College of Education at Qatar University had this to say “In our culture and our society, empathy, compassion, and kindness toward others are very important.”

#### 5.2.2 SWB, identity, and purpose in life

Dominant aspects of participants’ subjective identity narratives encompass their distinct identity as Muslims whose spiritual life is regulated by faith and religious values and principles. The analysis of the narratives further revealed a sense of purpose in life that is derived from spiritual beliefs and experiences, a connection that was stressed in the accounts reported by participants. Personal identity may be said to represent a person’s attributes, beliefs, values, and experiences, all of which are key to defining her/his purpose in life. With that said, the values, beliefs, and principles one embraces are primal features of one’s identity and, together with other factors, play a major part in defining one’s purpose in life.

Identifying themselves as Muslims, participants noted that having a sense of purpose in life provides a frame for their identity and well-being, as the narrative extracts below illustrate:

Participant 8 (male faculty, College of Education, Qatar University):

SWB for me is the desire to learn and acquire knowledge. Learning, as I see it, is not limited to earning a degree but goes beyond. Its curiosity and passion to learn, as asserted by the first word revealed in the Quran urging us to read “Recite (read) in the name of your Lord who created [humans].” Clear goals must therefore be set to chart the way to achieve these goals. So, the spiritual foundation for achieving well-being means setting goals identified by spirituality and aligning those goals to the correct path.

Participant 21 (male faculty, College of Arts and Sciences, Qatar University):

SWB is dynamic. It reflects our experiences of becoming who we are. We want to be seen and feel accepted … We need to have a direction in life and feel a sense of purpose. In the field of education, we have to cultivate these things. Creating a safe space and opportunities for experience, we have to know who we are and who we want to become.

Participants’ expressions of their religious identity demonstrate a sense of belonging to a spiritual community of like-minded people (fellow Muslims). From a discourse perspective, the frequent use of “we” above and “our” in the extract below lends evidence of a shared spirituality and may signify a collective, group, or plural identity, with evident expressions of similar beliefs, customs, and traditions. Other extracts from the narratives suggest that perceptions of SWB are linked to a sense of purpose in life and that the latter contributes to the development of an individual’s identity. According to Participant 11, a male faculty at the College of Business and Economics, Qatar University.

The spiritual grounds for achieving well-being mean two things to me. The first is to have a purpose that enriches spirituality, and the second is to fulfill that purpose by following the correct path of our religion.

Participant 21, a male faculty at the College of Arts and Sciences, Qatar University, viewed SWB in terms of “having life purposes that align with human nature” which, according to them, is “regulated by Shari’ah [Islamic law].” Accounts by other participants indicated that setting goals in life is closely intertwined with a person’s identity, hence stressing the value of “understanding our purpose in life” and highlighting the important questions related to her/his existence.

Our findings highlight the significance of socio-cultural factors in shaping faculty members’ perceptions and experiences of SWB. Socio-cultural theory provides a useful framework for understanding how faculty identities and experiences are shaped by the intersection of field-specific cultures, institutional environments, and broader societal values. This approach is particularly relevant when examining how subjective well-being (SWB) is perceived and experienced by faculty members in the context of Qatar. Our study supports the idea that any exploration of SWB in such contexts must acknowledge the significant role of religious and cultural factors, which play a central role in shaping faculty members’ experiences in higher education.

SWB, as a multidimensional concept, is closely linked with religiosity and adherence to Islamic values, which are integral to the socio-cultural fabric of Qatar. These values reflect the broader cultural context, where spirituality and religiosity play significant roles in defining both personal and professional identities. In an Arab-Muslim context like Qatar, institutional environments are largely shaped by Islamic values and community norms, which emphasize social cohesion and moral rectitude.

The distinction between private and public universities is not only economic but also cultural. Each institution type has distinct values, priorities, and organizational structures that influence faculty experiences. By considering these socio-cultural factors, we gain a better understanding of how faculty members manage their professional and personal lives within the framework of their spiritual beliefs and community expectations. Our findings stress the importance of socio-cultural factors in shaping faculty members’ perceptions and experiences of SWB. This socio-cultural lens challenges traditional Western paradigms of well-being, highlighting the need for a nuanced understanding of SWB in Arab-Muslim societies.

## 6 Discussion and conclusion

This study underscores the importance of anchoring perceptions of subjective well-being (SWB) in people’s lived experiences, norms, values, and cultural contexts. As supported by existing literature, the concept of SWB is holistic, intertwined with emotional, intellectual, social, and cultural dimensions. It is therefore evident that SWB cannot be readily investigated in isolation from these factors. In line with this perspective, our study’s findings suggest that SWB is a complex and multi-dimensional concept that evolves over time and involves several interconnected aspects of a person’s life, including intellectual, physical, psychological, social, and spiritual dimensions (Vik and Carlquist, 2017).

The subjective nature of well-being makes it difficult to establish a singular, universally agreed-upon definition (Diener, 2009). Perceptions of SWB differ across diverse contexts and individuals, shaped by their unique experiences, values, and cultural backgrounds. As highlighted in the literature, this variability complicates the operationalization of SWB, making it fluid, dynamic, and difficult to define in concrete terms (Vik and Carlquist, 2017). Participants’ narratives in this study support these findings, illustrating the lack of a unanimous definition of well-being, which is influenced by a myriad of factors.

A person’s cultural background, education, personal beliefs, practices, religious affiliation, and exposure to diverse perspectives significantly influence how they perceive and define SWB. However, despite the richness of the views expressed in the narratives analyzed, they fail to fully capture the complex and nuanced nature of SWB. While the narratives illustrate various emotional, intellectual, psychological, and physical dimensions of SWB, they do not convey the subtle distinctions that the concept entails.

The findings of this study have important implications for promoting faculty well-being in Qatar. By recognizing the integral role of socio-cultural factors, particularly Islamic values and community norms, institutions can develop culturally sensitive support systems. Such systems would promote environments that encourage spiritual growth and adherence to Islamic ethics, key elements for enhancing SWB among faculty members. This approach not only supports individual well-being but also contributes to social cohesion and moral rectitude, aligning with Qatar’s cultural and religious framework.

Based on the analysis of the voices and narratives of the participants, it is evident that spirituality, at its core, is the fundamental essence of their experience and entails the search for and expression of meaning in life (Zamaniyan et al., 2016). As is stated in more detail below, spirituality describes one’s beliefs and faith as well as one’s search for meaning in life. Indeed, beliefs, values systems, traditions, and practices are all expressions of an individual’s spirituality. For example, spiritual activities, such as prayers, fasting and visits to places of worship, enhance one’s life satisfaction and lessen psychological distress (Estupiñan and Kibble, 2018).

Spirituality is a very personal experience in one’s life. Similarly, SWB represents a deeply personal journey that affects people’s overall well-being and quality of life. While there is no one-size-fits-all approach to developing SWB, educational institutions can play an important role in promoting empathy, ethical values, self-worth, and a sense of purpose among faculty, students, and staff members. These and other virtues need to be incorporated into curricula and programs that foster them. A holistic approach that emphasizes moral and ethical values and helps to enhance the SWB of those in higher education should be developed. Equally useful will be to use an interdisciplinary approach that integrates ethical, intellectual, and spiritual dimensions into various subjects, such as the arts, humanities, and sciences.

Demonstrably, accounts reported by participants highlight diverse aspects of SWB perceived as comprising what is personal (meaning, purpose, and value of life), communal (fairness, forgiveness, humility, love, morals, respect, trust, and values), and transcendental (the relationship with the Divine and worship and adoration of religion; Fisher, 2021). Nonetheless, participants’ reports fail to capture the environmental dimension (living in harmony with the surrounding environment including fauna and flora) and the physical dimension (exercise, sports activities, etc.), which are no less important. No doubt, as Oglesby et al. (2021) argue, spirituality can serve as a panacea for palliating many health woes, particularly given it involves the way a person establishes connections between themselves, others, the natural world, and the divine (Fisher, 2021).

Viewed as a form of discourse, participants’ subjective narratives provide insights into their identity and their SWB. These narratives were treated in this study as a form of discourse that reflects their personal contextualized beliefs, experiences, perspectives, and social realities (Palmer et al., 2010). Influenced by the local Arab-Islamic context of Qatar, the narratives may be interpreted as stories that reflect participants’ identity, spirituality, and SWB. While acknowledging the potential effect of cultural norms and values, memory bias, and social desirability, narratives were analyzed bearing in mind the institutional affiliations of the participants and the socio-cultural context of the study (Foster et al., 2011).

Attachment and reverence to the divine (Allah) and devotion to Islam in general demonstrate participants’ SWB. This was associated with several indicators of SWB, including the comfort obtained from their perceived psychological proximity to the divine and the depth of their relationship with Him. Turning to the divine has been reported in the literature as being associated with an improved and stronger sense of well-being (Granqvist and Kirkpatrick, 2008) and satisfaction and happiness in life and enjoyment of good health (Amiruddin et al., 2021). This further echoes the conclusion derived from past research, which reveals that religiosity and SWB are strongly correlated to happiness, life satisfaction, and mental and physical health (Abdel-Khalek, 2014a, 2019; Shahama et al., 2022).

Participants’ subjective narratives yielded perspectives on spirituality and religious beliefs that reflect their distinct identity as Muslims. Their stated sense of purpose in life is derived from their faith and religious principles. Islam is more than a religion; it is considered a comprehensive way of life in Muslim societies (Oplatka and Hassan, 2023). It governs various spheres of society (education, health, family relationships, welfare, etc.) and plays a significant role in shaping people’s religious identity and well-being (Ayub et al., 2022; Kapur et al., 2022). Findings from our study revealed that, regardless of their affiliation, participants’ perspectives on SWB are greatly influenced by their faith and religious beliefs.

A recurrent feature that emerged from the narratives is the call for adherence to ethical conduct, moral values, and righteous behavior rooted in the principles and teachings of Islam. Participants’ frequent references to the importance of virtues and ethical values and the need to uphold the ideals of integrity, honesty, respect, tolerance, etc. can be understood against the background of sweeping global and external forces. It could be argued that the phenomena of globalization and Westernization may have ushered in immense social, cultural, economic, and other influences affecting various spheres of life in Qatar’s conservative Arab-Islamic society. With the increasing inclination to emulate Western values under the pretext of modernity, a more extreme version of such fears is that these changes may result in abandoning own religious principles and forsaking values in society.

Participants generally displayed similar perspectives on the topic of SWB and its intimate interconnectedness with spirituality and religion (Islam). Overall, their views on SWB concur on the crucial role of Islam, which provides a framework or code of conduct that regulates values, ethics, and morality. Similarly, across our data, there was a notable consensus among the participants on how their religious beliefs, values, and principles reflect their spirituality and embody their identity as Muslims. As revealed in our analysis, the faculty’s spiritual beliefs and experiences are key influencers that give them meaning and a sense of purpose in their lives.

Against the researchers’ expectations, participants’ institutional affiliation did not emerge as a factor that determines the way college/university faculty defined and perceived SWB. Interestingly, available research demonstrates that different disciplines – and those specialized in these fields for that matter – may use different theoretical lenses, frameworks, and approaches to study well-being (Diener, 2009; Das et al., 2020; Rusk and Waters, 2015). For example, psychology may focus on subjective well-being, which includes life satisfaction and happiness, while public health may consider objective indicators of well-being, such as physical health and social indicators (Diener, 2009). Awareness of the different dimensions and the various perspectives of well-being will help to gain a better understanding of this concept and inform the development of relevant policies and interventions.

Moreover, Islamic teachings offer a holistic approach to understanding well-being, emphasizing the interconnectedness of spiritual, moral, physical, emotional, and social dimensions. The Qur’an provides guidance on mental health, describing *Nafs* (the self) in three states that reflect the soul’s journey toward spiritual and psychological well-being (Al-Abdulrazak and van Nieuwerburgh, 2024). For instance, the verse “Verily, in the remembrance of Allah do hearts find rest” (Qur’an 13: 28) highlights the role of spirituality in achieving mental peace and tranquility (McLaren et al., 2021).

Building on these spiritual foundations, Islamic psychology further expands on the interconnectedness of the different dimensions of well-being, offering a comprehensive framework for maintaining mental health. Islamic psychology, rooted in Quranic ethics, offers a holistic approach to mental well-being. It emphasizes the importance of community and social connections, recognizing the role of familial and social support structures in fostering mental health (Mursidin, 2023). Islamic psychology integrates principles such as *Taqwa* (mindfulness), self-reflection, and spiritual growth, providing a framework for developing resilience and coping skills (Mitha and Adatia, 2016). The concept of *Fitrah* (human nature) guides individuals toward a balanced life by connecting them with their Creator (Kharchoufa, 2024).

While Islamic psychology provides a broad framework for mental health, Sufism, with its focus on spirituality and inner transformation, offers unique practices that further enhance mental and emotional well-being. Sufism, a mystical dimension of Islam, contributes significantly to mental health and well-being. Sufi practices such as *Dhikr* (remembrance of Allah) and *Fikr* (deep reflection) are akin to mindfulness, promoting self-awareness and emotional balance (Shafii, 1985). These practices help individuals develop a strong connection with the Divine, fostering inner peace and resilience (Shafii, 1985). Research on Sufi practices suggests that participants experienced reduced psychological stress and improved emotional well-being, highlighting the therapeutic potential of Sufism (Nizamie et al., 2013; O’Riordan, 1999).

One of the limitations of our study is its sole focus on participants’ perceptions of their own well-being (subjective well-being). Also, looking at objective well-being measures, such as their health, income, social status, and social relationships, etc. will enrich the study’s analysis and help to improve our understanding of the factors that influence individuals’ perceptions of their well-being. Furthermore, the use of a longitudinal study remains a valuable tool for gaining informed insights into the multifaceted and dynamic nature of well-being as it allows tracking changes characterizing people’s well-being over time.

Comparative analyses with neighboring countries can enhance our understanding of regional approaches to well-being, revealing both shared strategies and distinctive cultural and policy nuances. Future research will explore these international perspectives to provide an informed assessment of well-being initiatives in the Middle East. For instance, Saudi Vision 2030 includes the *Quality of Life Program*, which aims to improve well-being through cultural, recreational, sports, and tourism initiatives. While primarily designed to support economic diversification and lifestyle enrichment, this program echoes Qatar’s efforts to foster a more dynamic society and enhance overall well-being. Similarly, the UAE’s *National Strategy for Wellbeing 2031* offers a valuable point of comparison, as it prioritizes well-being at the individual, societal, and national levels through initiatives that promote healthy lifestyles, mental health, and positive thinking. Much like Qatar’s emphasis on spiritual well-being, the UAE’s strategy adopts a holistic perspective, integrating physical, mental, and social dimensions of well-being.

Based on the study’s findings, higher education institutions in Qatar should integrate SWB principles more explicitly into their faculty development programs, mental health initiatives, and work-life balance policies. Specifically, institutions could offer workshops on Islamic mindfulness techniques tailored for academics, establish spiritual counseling services within university counseling centers, and implement “Spiritual Wellness Days” allowing faculty time off for spiritual retreats or practices. Creating designated “Contemplation Spaces” on campus for prayer and reflection would also support faculty SWB.

An additional suggestion concerns developing an interdisciplinary approach that incorporates ethical, spiritual, and intellectual dimensions into various academic subjects. This could involve creating a seminar series on “Spiritual Wellness in Academia” and establishing a mentorship program pairing new faculty with senior colleagues who can provide guidance on maintaining spiritual well-being in an academic context. By implementing these recommendations, institutions can foster an environment that respects and nurtures the spiritual dimension of faculty well-being, in consistency with the Islamic values and cultural context of Qatar.

## Data Availability

The original contributions presented in the study are included in the article/supplementary material, further inquiries can be directed to the corresponding author.
